# Enhancing Anti-Tumoral Potential of CD-NHF by Modulating PI3K/Akt Axis in U87 Ex Vivo Glioma Model

**DOI:** 10.3390/ijms22083873

**Published:** 2021-04-08

**Authors:** Gabriel Luta, Mihail Butura, Adrian Tiron, Crina E. Tiron

**Affiliations:** Regional Institute of Oncology, 2–4, General Henri Mathias Berthelot Street, 700483 Iasi, Romania; gabriel.luta@iroiasi.ro (G.L.); mihail.butura@iroiasi.ro (M.B.); transcendctiron@iroiasi.ro (C.E.T.)

**Keywords:** glioblastoma, anti-cancer agent, carbon dots, EMT, nanomedicine

## Abstract

Background: In the latest years, there has been an increased interest in nanomaterials that may provide promising novel approaches to disease diagnostics and therapeutics. Our previous results demonstrated that Carbon-dots prepared from *N*-hydroxyphthalimide (CD-NHF) exhibited anti-tumoral activity on several cancer cell lines such as MDA-MB-231, A375, A549, and RPMI8226, while U87 glioma tumor cells were unaffected. Gliomas represent one of the most common types of human primary brain tumors and are responsible for the majority of deaths. In the present in vitro study, we expand our previous investigation on CD-NHF in the U87 cell line by adding different drug combinations. Methods: Cell viability, migration, invasion, and immunofluorescent staining of key molecular pathways have been assessed after various treatments with CD-NHF and/or K252A and AKTVIII inhibitors in the U87 cell line. Results: Association of an inhibitor strongly potentiates the anti-tumoral properties of CD-NHF identified by significant impairment of migration, invasion, and expression levels of phosphorylated Akt, p70S6Kinase, or by decreasing expression levels of Bcl-2, IL-6, STAT3, and Slug. Conclusions: Using simultaneously reduced doses of both CD-NHF and an inhibitor in order to reduce side effects, the viability and invasiveness of U87 glioma cells were significantly impaired.

## 1. Introduction

The brain, along with the spinal cord, forms the central nervous system (CNS) that controls most of our bodies’ activities. The brain is mechanically protected by the skull/cranium and chemically by the blood-brain barrier (BBB) that separates the brain from the general circulation. Brain cancers remain one of the challenging tumors to treat, characterized by high morbidity and mortality due to their localization and particularly locally invasive growth [1,2]. Gliomas represent one of the most common types of human primary brain tumors and are responsible for the majority of deaths [3]. According to their malignancy, gliomas are divided into four grades (WHO I–IV), less aggressive being astrocytoma (grade I—5–10 years average survival) and most malignant being glioblastoma (GMB, WHO grade IV—1, 5-year average survival) [4]. Standard treatment, consisting of surgery combined with radiotherapy and chemotherapy, is still inefficient due to the fact that GMB is characterized by a high degree of intra-tumor heterogeneity, strong microvascular proliferation, and infiltrating properties [5,6]. Molecular studies classified GBM into four subtypes like proneural (PN), neural (NL), classical (CL), and mesenchymal (MES), where the MES subtype is the most aggressive and strongly associated with a poor prognosis compared to the PN subtype. Moreover, in patients following radiation therapy and chemotherapy occur a conversion from PN to MES. The molecular cascades driving this conversion are similar to those driving the Epithelial-Mesenchymal Transition (EMT) in carcinomas cells [7,8]. The molecular mechanisms driving EMT consist of the down-regulation of epithelial specification genes like E-cadherin and up-regulation of mesenchymal markers such as vimentin and N-cadherin. Primary tumors that express mesenchymal markers are associated with enhanced invasiveness, therapy resistance, and poorer clinical prognosis [9,10]. EMT transcription factors such as SLUG, SNAIL, and TWIST have been shown to promote proliferation and invasion in both epithelial cancer cells but also in GBM [11,12,13].

Neurotrophins (NT), such as brain-derived neurotrophic factor (BDNF), nerve growth factor (NGF), neurotrophin (NT)-3, and NT-4/5 play important roles in the proliferation, differentiation, cell death in several cell types, including malignant cells. Neurotrophins mediate their functions through two distinct classes of transmembrane receptors: the p75 neurotrophin receptor (p75NTR), which binds to similar affinity all the neurotrophins, and tyrosine kinase receptors called tropomyosin related kinases (Trks) that display specificity in neurotrophin binding. It has been shown that p75NTR plays a critical role in the migration and infiltration of glioma cells into normal brain tissues [14]. p75NTR acts as a receptor inducing JNK-p53-Bax pathway activity or suppressing Ras/PI-3k/Akt activation while Trk receptors promote cell survival via different signaling cascades such as Ras/PI-3k/Akt pathway [15,16].

Improved molecular understanding of GBM, together with novel targeted agents, may contribute to personalized therapies [17,18]. Obstacles of successful treatment in the case of brain tumors comprise the impossibility of many drugs to penetrate the blood-brain barrier (BBB) [19,20].

Nanostructures, such as carbon dots (C-dots), are developed as drug delivery systems in order to improve solubility and accumulation of different drugs at the tumoral site, reduces drug side effects, and improve drug tolerance [21,22]. In the case of single drug administration, the drug applies their anti-cancer activity through a specific pathway, while combination drug therapy act in different pathways to increase the anti-cancer activity [23,24]. Anti-cancer activity of gemcitabine and fluorouracil (5FU) has been potentiated by curcumin [25,26]. P-Glycoprotein antibody functionalized Carbon Nanotubes (CNTs) loaded with doxorubicin significantly enhance the cytotoxicity of K262 leukemic cells compare with free doxorubicin [27]. Epidermal growth factor receptor (EGFR)-targeted polymeric nanoparticles (NPs) loaded with lonidamine and paclitaxel showed increased antitumor activity in human breast and ovarian tumor cells [28]. Carbon-dots conjugated with transferrin, epirubicin, and temozolomide had more antitumor efficiency compared with a single treatment combination in brain tumor cell lines [29]. Moreover, Carbon-dots are able to penetrate BBB [30]. Our previous results [31,32,33] demonstrated that anti-tumoral properties exhibited by Carbon-dots prepared from N-hydroxyphthalimide (CD-NHF) do not affect U87 brain tumor cells while significantly reduce cell viability and invasiveness of other representative cancer types, like breast cancer (MDA-MB-231, MCF7, and 4T1), melanoma (A375 and B16F10) or lung cancer (A549). In the present study, we expanded our investigation using different therapy combinations, aiming to sensitize U87 glioma cells to CD-NHF treatment by inhibiting key molecular pathways.

## 2. Results and Discussion

### 2.1. The Effects of Combination Therapy on U87 2D Cell Viability

Human glioblastoma (GBM), the most aggressive type of all brain tumors, is associated with poor prognosis and a narrow response to current therapies. Multiple pathways play important roles in glioma progression like epidermal growth factor (EGF), receptor tyrosine kinase (RTK) signaling pathways, including Ras/MAPK (MAP kinase), PI3-K (phosphoinositide 3-kinase)/Akt, PLC (phospholipase C), and p53 pathway [34,35]. Our previous results showed that CD-NHF (5%, 50 µg/mL) alone does not significantly impair the viability of the U87 cell line [31]. In the current study, we tested two inhibitors. One of the inhibitors, K252A, is a pan-Trk inhibitor as well as their downstream signaling, including PI3/Akt and Ca^2+^/calmodulin-dependent protein kinase II (CaMKII). The second inhibitor, AKTVIII, although it can inhibit all 3 Akt isoforms, is more specific for Akt1 and Akt2 and can also inhibit Ca2+/calmodulin-dependent protein kinase Iα (CaMKIα) [36]. Taking into consideration the associated toxicities of PI3K/Akt/mTOR inhibition [37] and the new data that demonstrates the increased metastasis induced by nanoparticles via inducing endothelial leakiness [38], we tested reduced concentrations of both CD-NHF and inhibitors.

**Figure 1:**
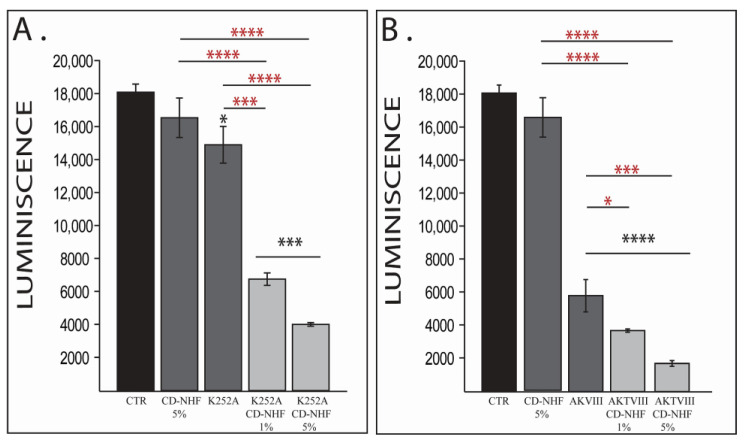
The effect of Carbon-dots prepared from *N*-hydroxyphthalimide (CD-NHF), K252A (**A**), and AKTVIII (**B**) inhibitors on U87 cell viability. Combined treatments have also been compared with their corresponding single treatment groups (red *). * *p* < 0.05; *** *p* < 0.0005; **** *p* < 0.00005.

To effectively assess the role of AKTVIII and TrkB inhibitors in CD-NHF U87 brain cancer cells, we first investigate their effect at the viability level (Figure 1).

As expected, and in line with our previous data, 5% CD-NHF (50 µg/mL) treatment did not significantly impair cell viability relative to the control group (Figure 1A,B). The presence of K252A or AKTVIII inhibitors alone significantly affected brain cancer cell viability (Figure 1A,B), AKTVIII exhibited a more pronounced effect relative to K252A. The increased impact of AKTVIII on cell viability when compared with K252A is expected, as AKTVIII inhibits phosphorylation of Akt isoforms induced by multiple pathways, while the used concentration of K252A inhibited only the signals mediated by the TrkA receptor. Tested concentrations of inhibitors together with 1% or 5% CD-NHF reduce even more brain cancer cell viability in a CD-NHF dose-dependent manner (Figure 1A,B). Cell viability was also significantly decreased when comparing combined treatments with individual treatments. Together, these findings indicate that combination therapy had a significantly higher effect on the viability of U87 cells (brain tumor cells) in 2D culture. These results demonstrate that tested inhibitors sensitize U87 cancer cells to the action of CD-NHF. In order to comply with the main hypothesis, for the next sets of experiments, we tested only 1% CD-NHF (10 µg/mL).

### 2.2. The Effects of Combination Therapy on U87 Cell Migration and Invasion

**Figure 2:**
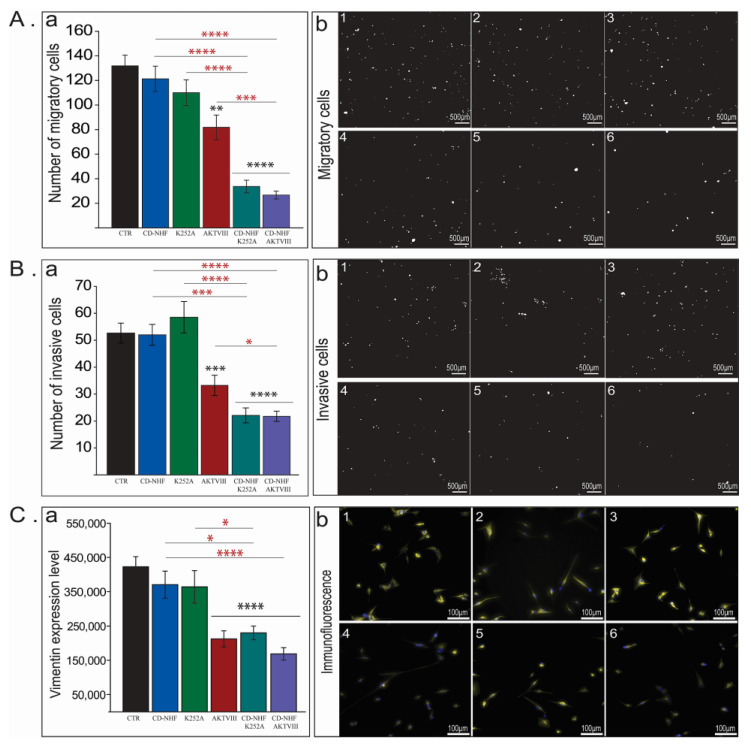
The effect of CD-NHF, K252A and AKTVIII inhibitors on U87 cell migration and invasion. (**A**) Cell Migration. (**a**) Quantification of migratory cells. (**b**) Representative images of migratory cells. (**B**) Cell Invasion. (**a**) Quantification of invasive cells. (**b**) Representative images of invasive cells (nuclei—white colour). (**C**) Vimentin expression levels of 2D cultured cells. (**a**) Quantification of vimentin. (**b**) Representative images (vimentin—yellow colour, nuclei—blue colour). (1). Control (untreated), (2). 1% CD-NHF, (3). K252A (5 nM), (4). AKTVIII (1.7 µM), (5). K252A + 1% CD-NHF, (6). AKTVIII + 1% CD-NHF. Pictures acquired at 10× (**A** and **B**) and at 20× ©. Scale bars are 500 µm (**A** and **B**) and 100 µm (**C**). * *p* < 0.05; ** *p* < 0.005; *** *p* < 0.0005; **** *p* < 0.00005.

Due to the fact that invasive glioblastoma cells represent a major cause of tumor relapse and mortality, we assessed the effect of combination therapy on brain cancer cell invasiveness (Figure 2).

The presence of 1% CD-NHF or K252A (Figure 2A,B) did not significantly impair the migration or invasion of U87 cells compared to control. The second tested inhibitor, AKTVIII (Figure 2A,B) significantly impaired both the migration and invasion of U87 cells, and this could be correlated with a more pronounced inhibition of cell viability relative to K252A treatment. Moreover, combined treatments (CD-NHF and an inhibitor) strongly inhibited migration and invasion for both tested inhibitors (Figure 2A,B; 5,6). Migration and invasion are significantly decreased also when comparing combined treatments with the individual treatments. The effect of combined treatments on migration and invasion complement their effect on cell viability. When comparing treated groups with the control group, immunofluorescence staining with vimentin (Figure 2C), a marker of EMT, supports the migration and invasion data by demonstrating the downregulation of the EMT marker in the same treated groups in which the migration and invasion were found to be significantly impaired. However, vimentin expression in CD-NHF and AKTVIII combined treatment was significantly reduced only relatively to CD-NHF treatment. This may be due to the fact that AKTVIII alone, at the tested concentration, is efficient in inhibited vimentin expression, and the combined treatment does not decrease vimentin expression under a certain basal expression.

### 2.3. The Effects of Combination Therapy on U87 3D Matrigel Assay

**Figure 3:**
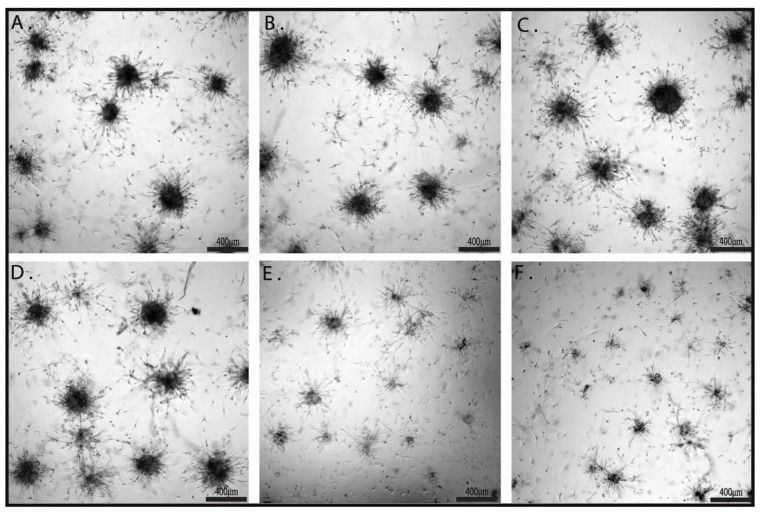
The effects of CD-NHF, K252A, and AKTVIII inhibitors on U87 3D cell matrigel assay. (**A**) Control, (**B**) K252A, (**C**) AKTVIII, (**D**) CD-NHF, (**E**) K252A + CD-NHF, (**F**) AKTVIII + CD-NHF. Pictures acquired at 5× magnification. Qualitative analysis reveals obvious morphological differences of spheroid appearances in terms of overall dimensions, geometric uniformity/complexity, protrusion arm extension and ramifications, and neighboring spaces tumor cell seeding efficiencies.

Cell viability/proliferation and invasiveness can be investigated at the same time in a higher-level cell culture system, a 3D culture system. Cells are cultivated in a matrigel that allows them to form spheroids. Inside of the bigger spheroids, starvation occurs, leading to apoptosis and the formation of microenvironmental niches. This reproduces cell interaction in a more complex way. The size of the spheroids reflects cell viability/proliferation, while invasive projections correlate with invasiveness. Brightfield microscopy investigation of formed spheroids (Figure 3) further proved the efficiency of combined treatment by both spheroid size reduction and by impairment of invasive projections. Fluorescence imaging of the spheroids is provided in Supplementary Figure S1.

In the 2D system, both inhibitors alone significantly impair cell viability while only the AKTVIII inhibitor significantly reduced cell migration and invasion. In the 3D system, treatments with inhibitors alone did not noticeable impair spheroids sized or branches. Only combined treatments visible reduced the spheroids sizes. This data further proves the efficiency of combined treatments.

### 2.4. The Effects of Combination Therapy on TrkB and P75NTR Receptors

**Figure 4:**
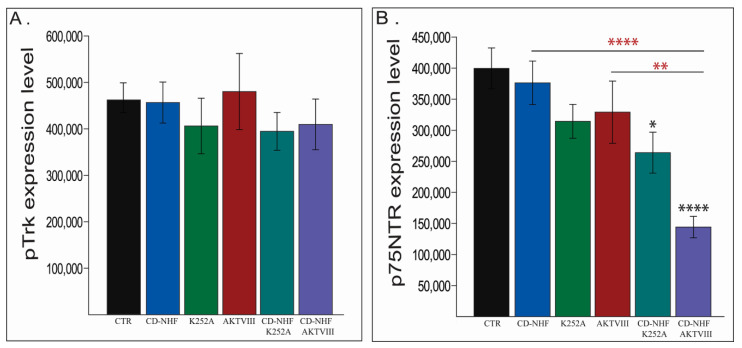
Assessment of TrkB phosphorylation and p75 neurotrophin receptor (p75NTR) expression levels. (**A**) TrkB phosphorylation. (**B**) p75NTR. Combined treatments has been also compaired with their corresponding single treatment groups (red *). * *p* < 0.05; ** *p* < 0.005; **** *p* < 0.00005.

According to the manufacturer’s instructions, 5 nM of K252A is the nominal concentration sufficient to block CaMKII and TrkA receptor activity but partially spares TrkB and TrkC receptors. In order to demonstrate that the 5 nM k252A concentration does not inhibit the activity of all Trk receptors, we assessed the level of TrkB phosphorylation (Figure 4A). Immunofluorescence data demonstrates that there is no significant variation of TrkB phosphorylation across the treated groups.

Another receptor, p75NTR, which is an important regulator of glioma invasion and which can bind with low-affinity Trk agonists, has been next investigated (Figure 4B) due to its complex role (pro-apoptotic vs. cell survival and migration). To our surprise, although we did not directly target p75NTR, combined treatments significantly impaired its expression (Figure 4B), which was more prominent in CD-NHF-AKTVIII group. These results may be due to the interactions between Trk receptors and p75NTR or as a consequence of overall decreased cell survival. Representative staining images are provided in Supplementary Figures S2 and S3. In glioma, p75NTR plays a central role in the regulation of invasion [14], and decreased expression of p75NTR in combined treatment groups was correlated with impaired migration and invasion we reported. Migration, invasion, and vimentin were also decreased in the AKTVIII group without being accompanied by a significant loss of p75NTR expression. This indicates that p75NTR was not the only molecule involved in these processes. Expression of p75NTR was also significantly decreased when comparing the CD-NHF and AKTVIII combined treatment with the corresponding individual treatments. More importantly, in another type of cancer, p75NTR is a characteristic of the mitotically quiescent cancer stem population [39] which increases chemoresistance. If this also occurs in U87 cells, impairment of p75NTR expression may be associated with decreased chemoresistance.

### 2.5. The Effects of Combination Therapy on Downstream Trk Signalling Pathways

**Figure 5:**
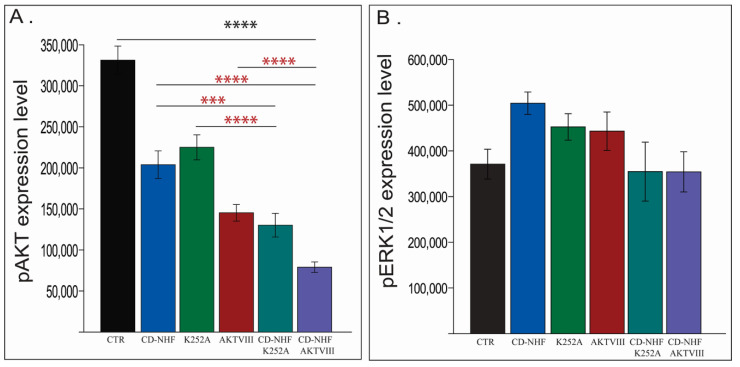
Assessment of Akt and ERK1/2 phosphorylation levels. (**A**). Akt phosphorylation. (**B**) ERK1/2 phosphorylation. Combined treatments compared with their corresponding single treatment groups (red *). *** *p* < 0.0005; **** *p* < 0.00005.

Trk receptors activate several molecular cascades, including PI3K/Akt, MAPK/ERK, and PLCγ pathway. In addition to K252A/Trk inhibition, the PI3K/Akt pathway can be inhibited directly by AKTVIII. The K252A used working concentrations do not inhibit all three Trk receptor types, while the AKTVIII inhibitor inhibited only Akt1/Akt2 subtype without inhibiting the Akt3 isoform. Our data (Figure 5A) demonstrates a significantly decreased phosphorylation level of pan-pAkt not only in K252A and AKTVIII treated groups but also in the CD-NHF treated group, relative to the control group. Moreover, the decrease in phosphorylation levels was even higher by combined treatments (Figure 5A), even when compared with their corresponding individual treatments.

The MAPK/ERK pathway (Figure 5B) assessed by measuring phosphorylation levels of ERK1/2 did not significantly adjust the expression levels between treated groups, suggesting that CD-NHF did not strongly interfere with this molecular pathway. Representative images are provided in Supplementary Figures S4 and S5. Our data indicate a non-significant increase in ERK1/2 phosphorylation levels in CD-NHF, K252A, and AKTVIII groups, suggesting that this pathway may be partially activated in compensation to inhibition of the PI3K/Akt pathway. This may explain why we did not observe a decrease in cell viability in the CD-NHF treated group. At the tested K252A concentration, the PLC/CaMKII axis should have been inhibited, as CaMKII represents the primary target of K252A. In glioma, CaMKII may contribute to cell proliferation [40], and repressing the PLC/CaMKII signaling by K252A may explain cell viability decrease observed in the K252A treated group, although pAKT and pERK1/2 had a similar pattern as in the CD-NHF group.

### 2.6. Other PI3K/Akt Downstream Targets

**Figure 6:**
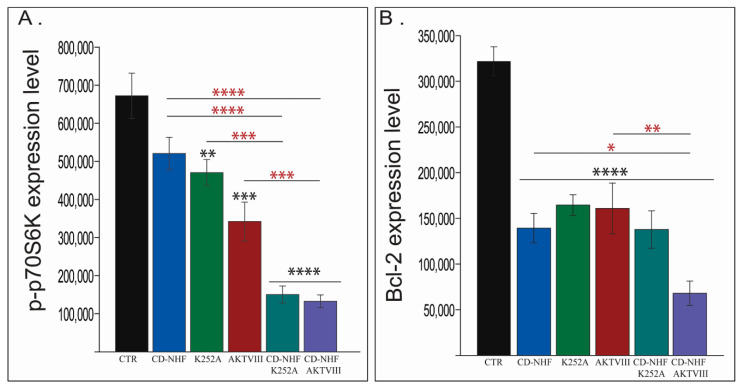
Assessment of p70S6 kinase (p70S6K) phosphorylation and Bcl-2 expression levels. (**A**) p70S6K phosphorylation. (**B**) Bcl-2. Combined treatments compared with their corresponding single treatment groups (red *). * *p* < 0.05; ** *p* < 0.005; *** *p* < 0.0005; **** *p* < 0.00005.

P70S6 kinase represents another key member of the PI3K/Akt/mTOR molecular cascade and plays a key role in protein synthesis, cell survival, and cell growth [41]. Our experimental data (Figure 6A) identified a non-statistically significant decrease in p70S6 kinase phosphorylation in CD-NHF and a significant reduction in both K252A and AKTVIII treated groups (Figure 6A). In addition, the decreased phosphorylation was found even higher in combined treatment groups compared with the control group (Figure 6A) and single treatment groups. Decreased p70S6 kinase phosphorylation correlated with impairment of cell viability and further proves the efficiency of the combined treatments.

Next, Bcl-2 (Figure 6B), a pro-survival molecule belonging to the Bcl family, representing another molecule from the PI3K/Akt molecular cascade, was investigated. The immunofluorescence staining revealed that Bcl-2 expression levels were significantly lower in all treated groups relative to the control group (Figure 6B. This impairment was at a higher magnitude in the CD-NHF-AKTVIII group and is significant even when compared with corresponding individual treatments. Representative images are provided in Supplementary Figures S6 and S7. The Akt/Bcl-2 axis was significantly impaired in CD-NHF treated group as demonstrated by assessing both Akt phosphorylation and Bcl-2 expression levels; however, cell viability was not impaired in this treated group. This could be due to the fact that Akt/Bcl-2 axis inhibition did not have the required magnitude to efficiently reduce pro-survival signals or due to pro-survival signals from other molecular pathways that may compensate for the impairment of the Akt/Bcl-2 axis in the CD-NHF treated group.

**Figure 7:**
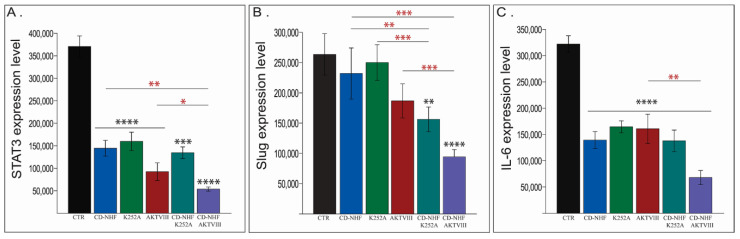
Assessment of STAT3, Slug and IL-6 expression levels. (**A**) STAT3. (**B**) Slug. (**C**) IL-6. Combined treatments compared with their corresponding single treatment groups (red*). * *p* < 0.05; ** *p* < 0.005; *** *p* < 0.0005; **** *p* < 0.00005.

Another class of molecules that are activated by tyrosine kinases, including Trks, is represented by members of the STAT family. STAT3, a member of the STAT family, plays a critical role in tumor cell proliferation, survival, and invasion [42]. Moreover, the STAT3/Slug axis has been reported to enhance cancer stem-like properties in radioresistant glioblastoma [43]. Under these circumstances, we next investigated the expression levels of STAT3 (Figure 7A) and Slug (Figure 7B).

Our data demonstrate significant impairment in the expression levels of STAT3 in all groups relative to the control group (Figure 7A). This impairment further demonstrates a higher magnitude in the CD-NHF-AKTVIII group, which was significant even in corresponding individual treatments (Figure 7A). Slug expression, although decreased in all treated groups, was statistically significant only in the combined treatment groups (Figure 7B) both versus the control group and versus corresponding individual treatments. Furthermore, STAT3 is activated in response to various stimuli, in addition to tyrosine kinases. Interleukin 6 (IL-6), a pro-inflammatory cytokine, mediates pro-survival signals in glioma stem cells through STAT3 [44]. Our data show a significant impairment of IL-6 levels in all treated groups when compared with an untreated control group (Figure 7C). In the CD-NHF-AKTVIII combined group, IL-6 expression was also significant relatively to AKTVIII alone treatment. Representative images are provided in Supplementary Figures S8, S9m and S10.

**Figure 8:**
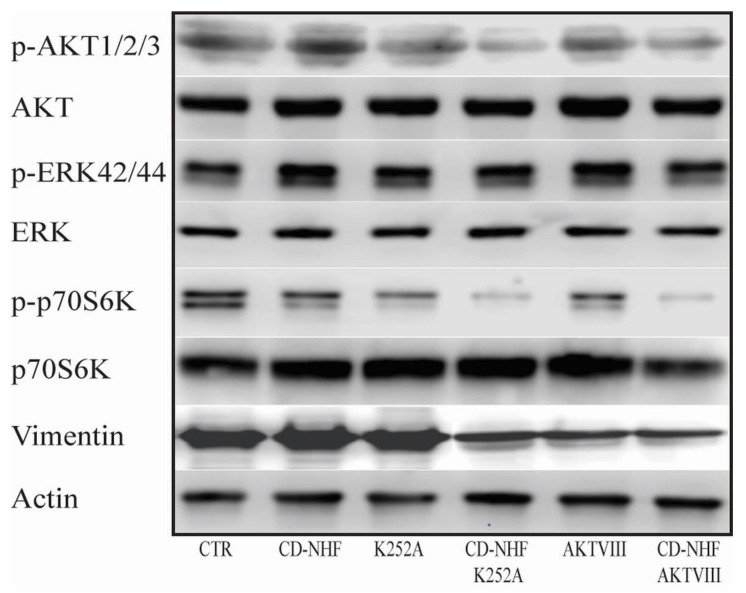
Western blot assessment of representative molecules in U87 cell line. Investigated molecules are key members of the two main molecular pathways, PI3K/Akt and MAPK/ERK, and for epithelial-mesenchymal transition (EMT).

Next, we assessed by Western blot (WB) the protein levels of Akt1/2/3, Erk42/44, p70S6K, and vimentin (Figure 8). While expression levels of total Akt, Erk, and p70S6K was not affected, their phosphorylation levels support the observersations of the immunofluorescence data. The vimentin WB data also reproduced the results from immunofluorescence.

At this moment, we do not have data about the exact mechanism of CD-NHF action. It seems to interact with various molecules downstream or upstream of receptors rather than interacting directly with receptors. It may bind to the molecules, which would prevent them from exerting their interactions fully with their partners. Neurotrophins and IL-6 mediate their downstream effects by activating similar molecular pathways: PI3K/Akt, MAPK, and JAK/STAT pathways [45,46,47]. We did not measure neurotrophins levels; however, phosphorylation of TrkB and the expression levels of p75NTR did not vary significantly in the CD-NHF group relative to the untreated control group. On the other hand, IL-6 expression was downregulated by CD-NHF. Downstream of activated receptors, CD-NHF significantly interacted with the AKT/Bcl-2 and Jak/STAT3 axes. Partial inhibitions of Trks or of PI3K/Akt potentiate CD-NHF effects on these pathways, as revealed by further impairment of expression levels of mentioned targets and by decreasing the phosphorylation of p70S6 kinase and Slug.

## 3. Materials and Methods

### 3.1. Cell Cultures

U87 cells (American Type Culture Collection, Rockville, MD, USA, ATCC), a generous gift from James Lorens (Bergen Bio AS, Bergen, Norway), were cultured in a humidified atmosphere at 37 °C with 5% CO2, in Eagle’s Minimum Essential Medium supplemented with 100 U/mL of penicillin, 100 μg/mL of streptomycin and 10% bovine serum (Sigma-Aldrich, St. Luis, MO, USA). 5000 cells per well were seeded in 96-well flat-bottom tissue culture plates for the 2D culture system. CD-NHF, K252A, and AKTVIII stock solutions have been diluted in growth medium to the tested concentrations. Control groups have been incubated with medium alone, without tested compounds. U87 Cell line was purchased from ATCC in August 2016 by Bergen Bio AS, Bergen, Norway and we received them in October 2016. Cells were kept in liquid nitrogen before we used them for experiments included in the current manuscript.

### 3.2. Cell Viability

For cell viability estimation, we used the CellTiter-Blue^®^ Cell Viability Assay (Promega, Madison, WI, USA). Following cell attachment, the cells were incubated with the CD-NHF at 5% concentrations (50 µg/mL), K252A (5 nM) (Sigma-Aldrich, 420297, St. Luis, MO, USA), AKTVIII (1.7 µM) (Sigma-Aldrich, 124018, St. Luis, MO, USA), CD-NHF-K252A, or CD-NHF-AKTVIII for 72 h. In combined treatments we tested both 1% (10 µg/mL) or 5% CD-NHF (50 µg/mL). After each of the 72 h treatment time periods, 50 μL of cell viability solution was added to each well, and the plate was reincubated for 4 h before fluorescence recording using a multiple microplate reader (FilterMax F5, Sunnyvale, CA, USA).

### 3.3. Migration and Invasion Assay

The assays were carried out using Cytoselect 24-well cell migration and invasion assay according to manufacture instructions (Cell Biolabs, Inc. CBA-100-C, San Diego, CA, USA). Cells were seeded at 5 × 105 cells per well in a serum-free medium supplemented with 0.1% BSA. The U87 cells were induced to migrate or invade toward medium containing 10% FBS alone or with 1% CD-NHF (10 µg/mL), K252A (5 nM), AKTVIII (1.7 µM), CD-NHF-K252A or CD-NHF-AKTVIII for 24 h in the CO2 incubator. Treated groups contained, in the inserted medium, the same treatment as in wells. Twenty-four hours post-seeding, non-invading cells were removed with a cotton swab. The remaining cells were fixed, stained with DAPI, and analyzed by fluorescence microscopy (10× magnification; Zeiss Axio Observer Z.1 microscope, TissueGnostics rig, Vienna, Austria). Sixteen images were acquired per well using TissueFAXS 4.2 software (Vienna, Austria) and quantified by using ImageJ software.

### 3.4. D Matrigel Assays

The 3D Matrigel assays were conducted with 1000 cells seeded in Ibidi plates between 2 layers of Matrigel (BD Matrigel Matrix, Growth Factor Reduced (BD Biosciences, Bedford, MA, USA)) and cultured for 14 days before microscopy analysis (TissueGnostic rig, Vienna, Austria). Twelve hours post-seeding, 3D embedded cells began to be treated with 1% CD-NHF, K252A, AVTIII, or combined treatment (CD-NHF and one inhibitor). After 72 h, the treatments were removed and replaced with normal 3D Matrigel medium (medium corresponding to U87 cell type supplemented with 2% fetal bovine serum (FBS) and 1% Matrigel (BD Biosciences, Bedford, MA, USA)). Nuclei were counterstained with NucBlue Live Ready Probes Reagent (Thermo Fisher Scientific, Eugene, OR, USA). Brightfield phase-contrast 1 and fluorescence pictures were acquired at 5× magnification using a Zeiss Axio Observer Z1 brightfield/fluorescence microscope from TissueGnostics rig (Vienna, Austria). Single focal plane images were acquired using Tissue FAXS 4.2 software.

### 3.5. Immunofluorescence Staining

At the end of the experiments, cells were fixed in 4% paraformaldehyde for 60 min. After permeabilisation with 0.3% Saponin (55825-100GM, Merck Millipore^®^), 0.3% Triton (Sigma-Aldrich, St. Luis, MO, USA) and 0.3% digitonin for 1 h, cells were incubated at 4 °C for 72 h with primary antibodies: anti-vimentin clone V9 (M0725, Dako^®^), 1:20; anti-pTrkB (phospho Y515, ab131483, Abcam^®^, Cambridge, UK), 1:25; anti-p75NTR (sc-271708, Santa Cruz Biotechnology^®^), 1:20; anti-pAkt1/2/3 (sc-271966, Santa Cruz Biotechnology^®^, Heidelberg, Germany), 1:20, anti-pERK1/2 (sc-136521, Santa Cruz Biotechnology^®^, Heidelberg, Germany), 1:20, anti-p-p70S6K (sc-8416, Santa Cruz Biotechnology^®^, Heidelberg, Germany), 1:20, anti-Bcl-2 (sc-7382, Santa Cruz Biotechnology^®^, Heidelberg, Germany), 1:20, anti-STAT3 (sc-8019, Santa Cruz Biotechnology^®^, Heidelberg, Germany), 1:20, anti-Slug (sc-166476, Santa Cruz Biotechnology^®^), 1:20, and anti-IL-6 (sc-130326, Santa Cruz Biotechnology^®^, Heidelberg, Germany), 1:20. These antibodies were diluted using an antibody dilution solution containing 0.3% saponin, 0.3% Triton X 100 and 0.5% goat serum. Alexa Fluor 647-conjugated goat anti-mouse IgG (A21241, ThermoFisher Scientific^®^, Waltham, MA, USA), 1:200 were applied overnight at 4 °C to respectively recognize the IgGs. Fluorescent dyes were protected by using ProLong Gold Antifade Mountant with DAPI (P36941, ThermoFisher Scientific^®^ Waltham, MA, USA) according to manufacture recommendations. p-Akt1/2/3 Antibody (B-5) detect Thr 308 phosphorylated Akt1, correspondingly Thr 309 phosphorylated Akt2 and correspondingly Thr 305 phosphorylated Akt3. Nuclei were used to identify each cell for quantitative analysis. Pictures were acquired at 20× with a Zeiss Axio Observer Z1 Microscope TissueGnostic using Tissue FAXS 4.2 software (Vienna, Austria). TissueQuest 6.0 software was used for quantitative cell analysis and to quantify the sum intensity of the fluorescence signal for each event.

### 3.6. Western Blot

Protein levels in homogenate samples were determined using the Bradford method. Equal amounts of protein (35 µg/well) were loaded onto SDS-PAGE gels (10%) and run at constant 100 V. Separated proteins were transferred to a nitrocellulose membrane (Sigma-Aldrich Whatman Protran nitrocellulose transfer membrane, St. Luis, MO, USA) at a constant voltage 100 V for 1 h. Membranes were blocked on a gyro-rocker for 1 h at room temperature (RT). The blocking buffer (BB) consisted of TBST (Tris-buffered saline/0.1% Tween 20) and 5% BSA or 5% non-fat dry milk. The primary antibodies were dissolved in BB containing 3% BSA or 3% milk (dilution 1:250 and 1:500), and the blots were incubated at 4 °C overnight with constant shaking. After three washes with TBST, blots were incubated for 1 h at RT in horseradish peroxidase-conjugated secondary antibody dissolved in TBST (dilution 1:5000). The blots were washed three times with TBST, and proteins were visualized using WesternSure PREMIUM Chemiluminescent Substrate (Li-Cor, Lincoln, NE, USA) and Image Studio Digits software provided by Li-Cor (Lincoln, NE, USA). Blots were stripped with WesternSure ECL Stripping Buffer (Li-Cor, Lincoln, NE, USA) at RT for 15 min and reprobed with another antibody detecting the protein of interest.

### 3.7. Statistics

GraphPad Prism was used for statistical analysis. Grouped analyses were performed by one-way ANOVA. Quantitative data for statistical analysis were expressed as mean ± SEM (shown as error bar). During the statistical analyses, we compared each treated group with the control group. The black asterisk represents the significance of the treated group relative to the control group. The significance of combined treatments with corresponding single treatment is indicated by the red asterisk above the bar. Significance was established using a threshold of *p* < 0.05.

## 4. Conclusions

Anti-tumoral properties exhibited by CD-NHF were potentiated by reduced doses of K252A or AKTVIII inhibitors in order to avoid side effects. More importantly, this potentiation was significant even at a reduced concentration of CD-NHF. Although we do not know the exact mechanism of CD-NHF action, our findings support the potential of CD-NHF as glioma-targeting pharmacologic agents.

## Supplementary Materials

The following are available online at https://www.mdpi.com/article/10.3390/ijms22083873/s1, Representative images for immunofluorescence spheroids staining (Figure S1), pTrkB (Figure S2), p75NTR (Figure S3), pAkt (Figure S4), pERK1/2 (Figure S5), p-p70S6K (Figure S6), Bcl-2 (Figure S7), STAT3 (Figure S8), Slug (Figure S9), and IL-6 (Figure S10) and uncut blots.
