# Supplementary material for: Enhancing Anti-Tumoral Potential of CD-NHF by Modulating PI3K/Akt Axis in U87 Ex Vivo Glioma Model
Source: Int J Mol Sci. 2021 Apr 8;22(8):3873. doi: 10.3390/ijms22083873 (PMC8070499; doi:10.3390/ijms22083873)
Supplement: Supplementary file 1 [file ijms-22-03873-s001.pdf]

Supplementary Material: Enhancing anti-tumoral potential of CD-NHF by modulating PI3K/Akt axis in U87 *ex vivo* glioma model

Gabriel Luta, Mihail Butura, Adrian Tiron and Crina Elena Tiron

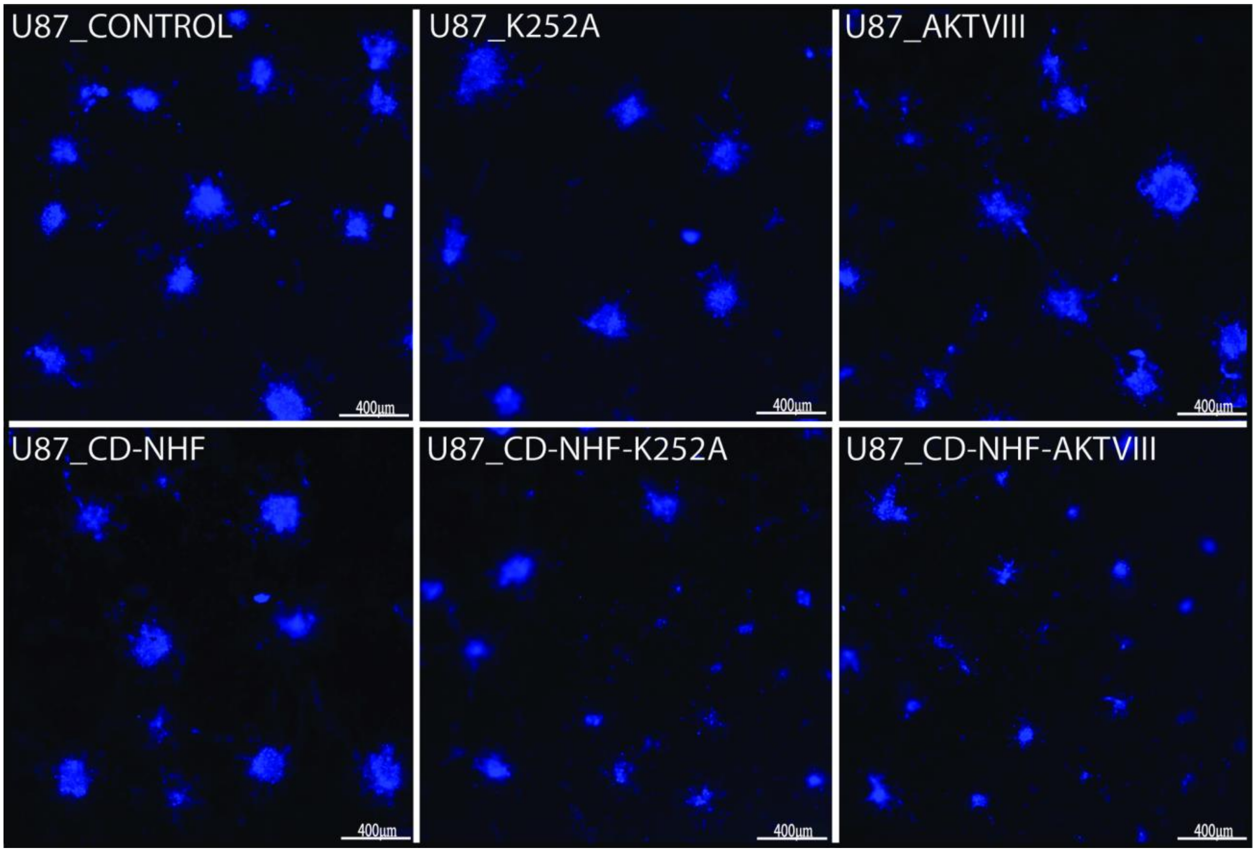

Figure S1. Representative images for immunofluorescence spheroids staining. Nuclei were stained with NucBlue Live Ready Probes Reagent. Pictures acquired at 5x.

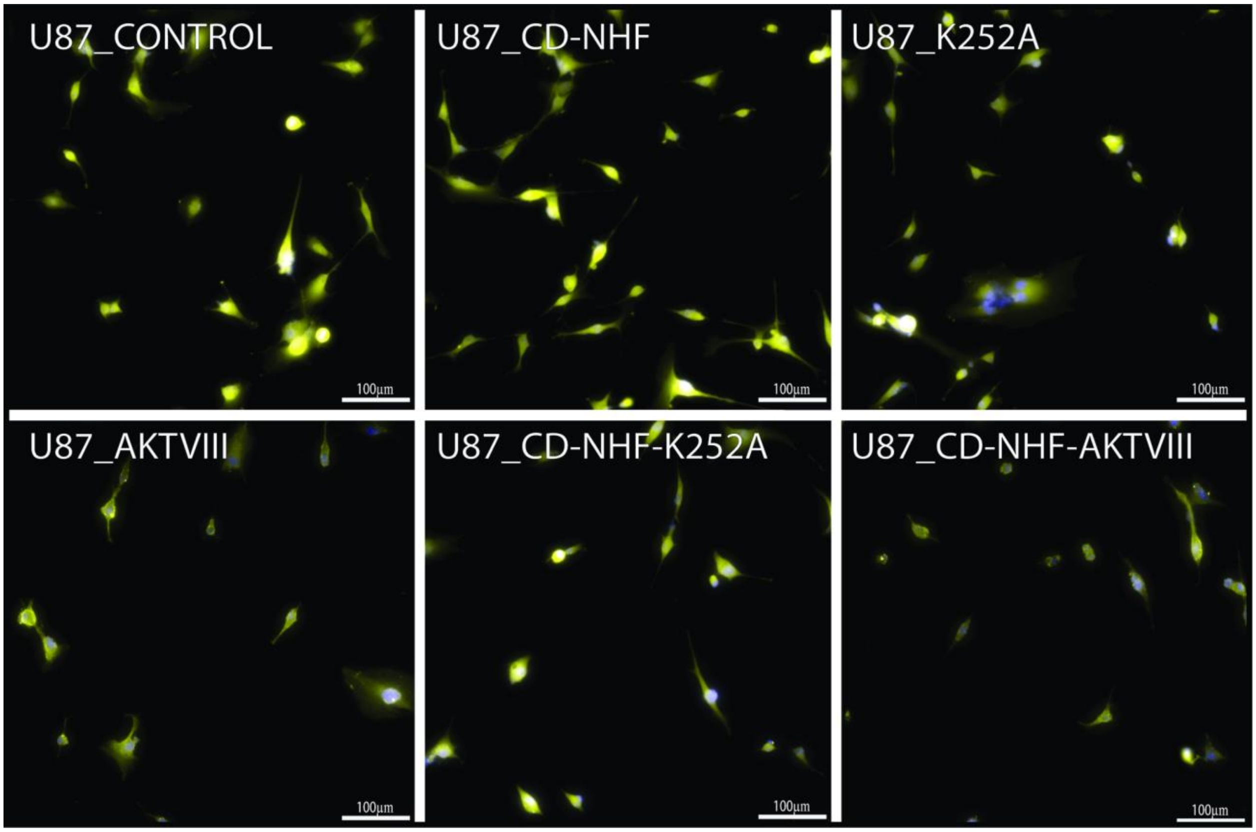

Figure S2. Representative images for immunofluorescence pTrkB staining. Merged stainings of phospho TrkB (yellow) and nuclei (blue). Pictures acquired at 20x.

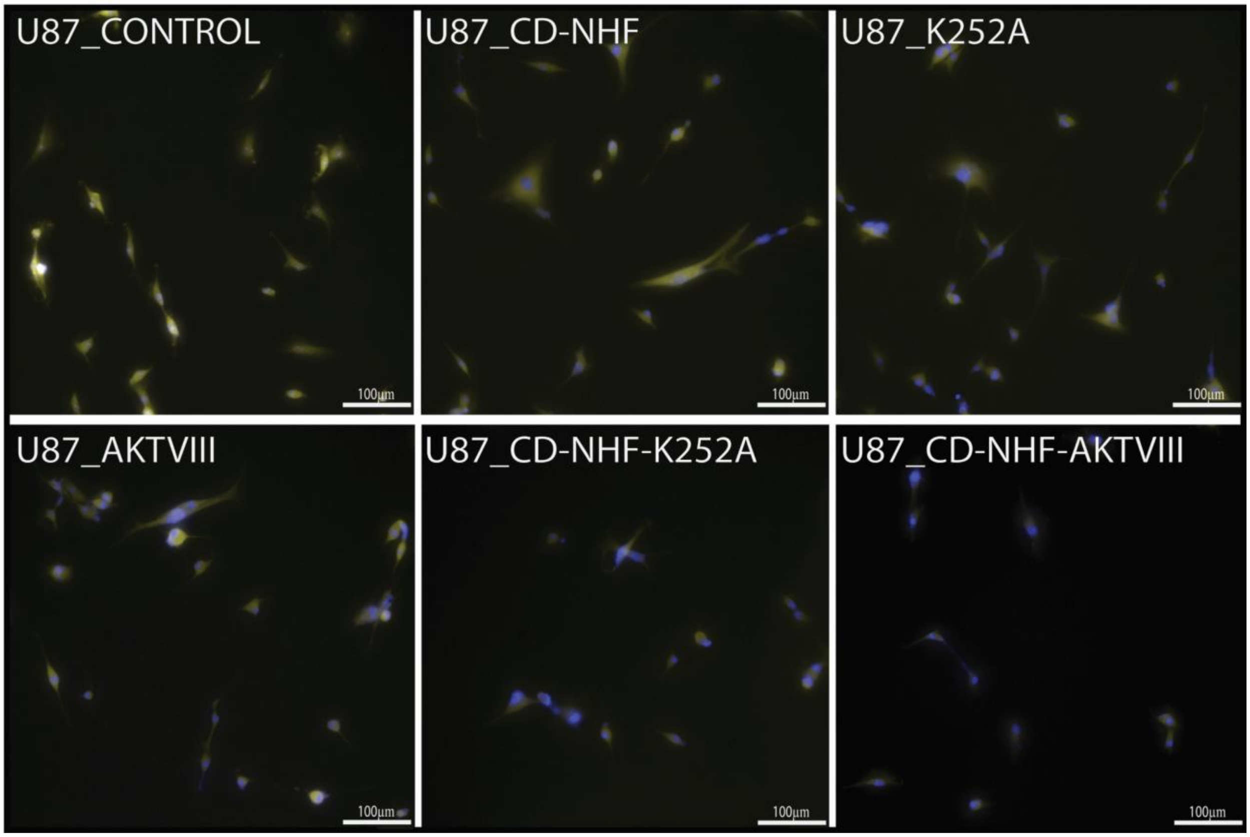

Figure S3. Representative images for immunofluorescence p75NTR staining. Merged stainings of p75NTR (yellow) and nuclei (blue). Pictures acquired at 20x.

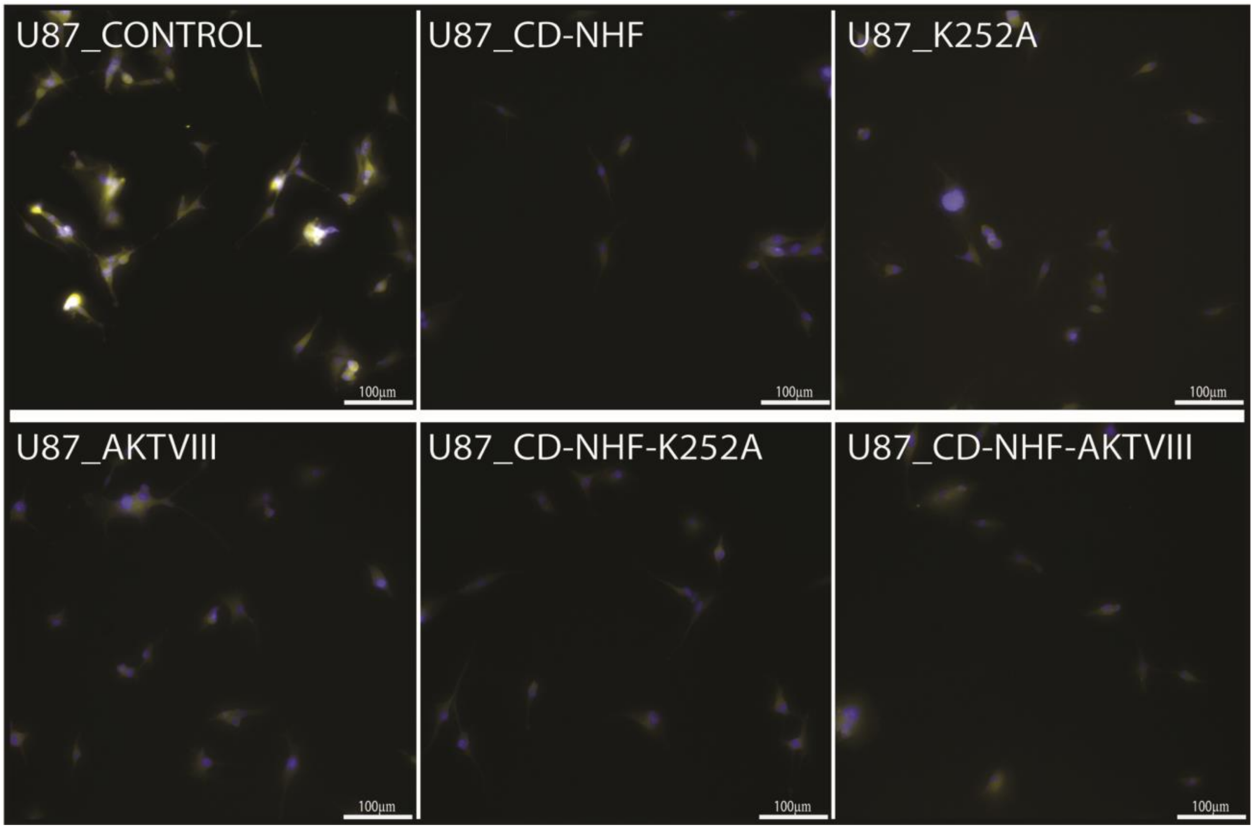

Figure S4. Representative images for immunofluorescence pAkt staining. Merged stainings of phospho Akt1/2/3 (yellow) and nuclei (blue). Pictures acquired at 20x.

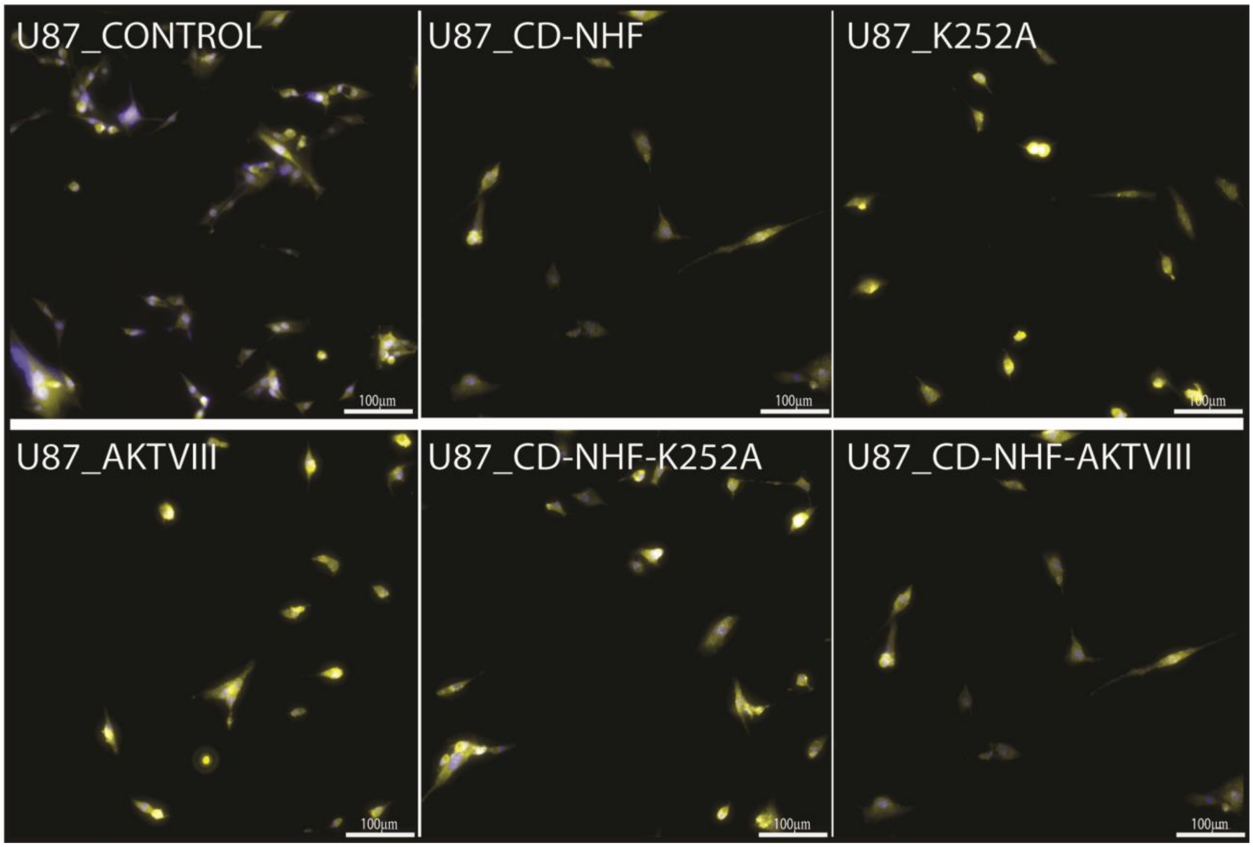

Figure S5. Representative images for immunofluorescence pERK1/2 staining. Merged stainings of phospho ERK1/2 (yellow) and nuclei (blue). Pictures acquired at 20x.

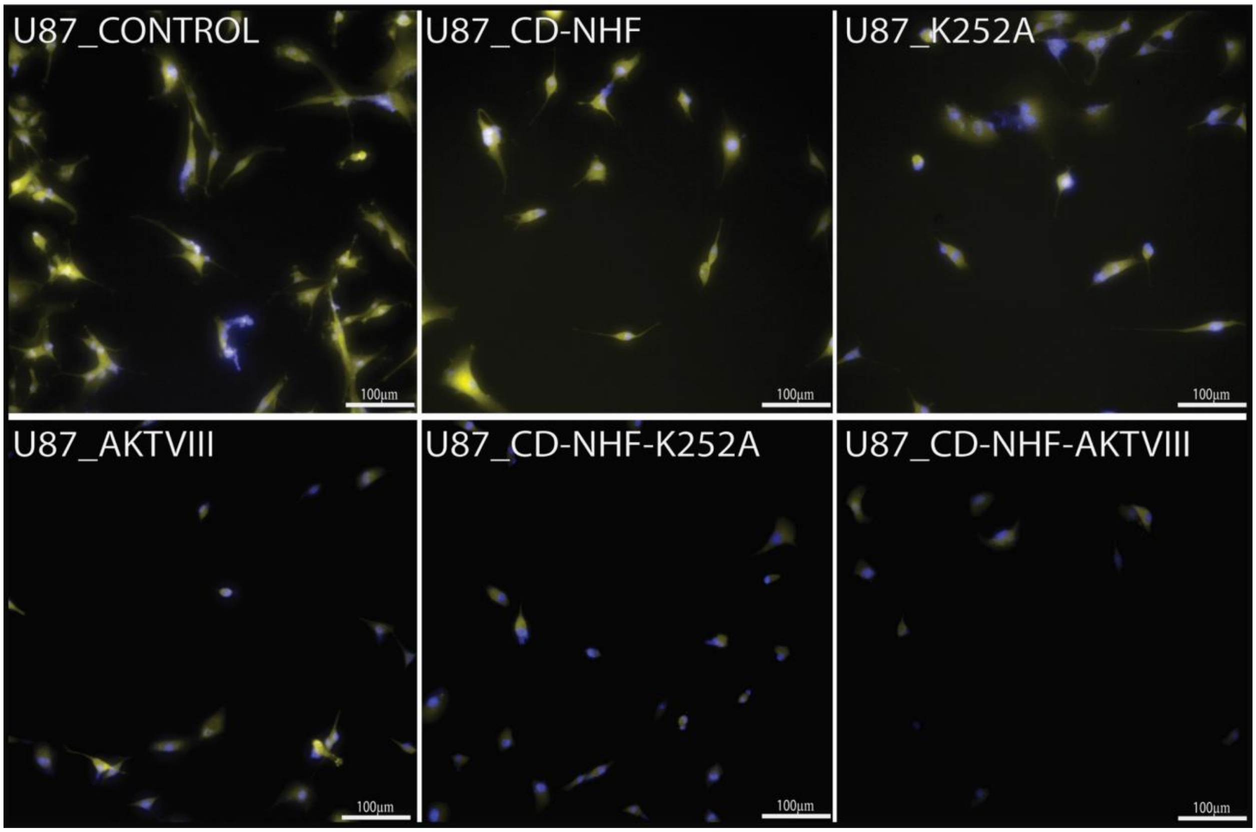

Figure S6. Representative images for immunofluorescence p-p70S6K staining. Merged stainings of phospho p70S6K (yellow) and nuclei (blue). Pictures acquired at 20x.

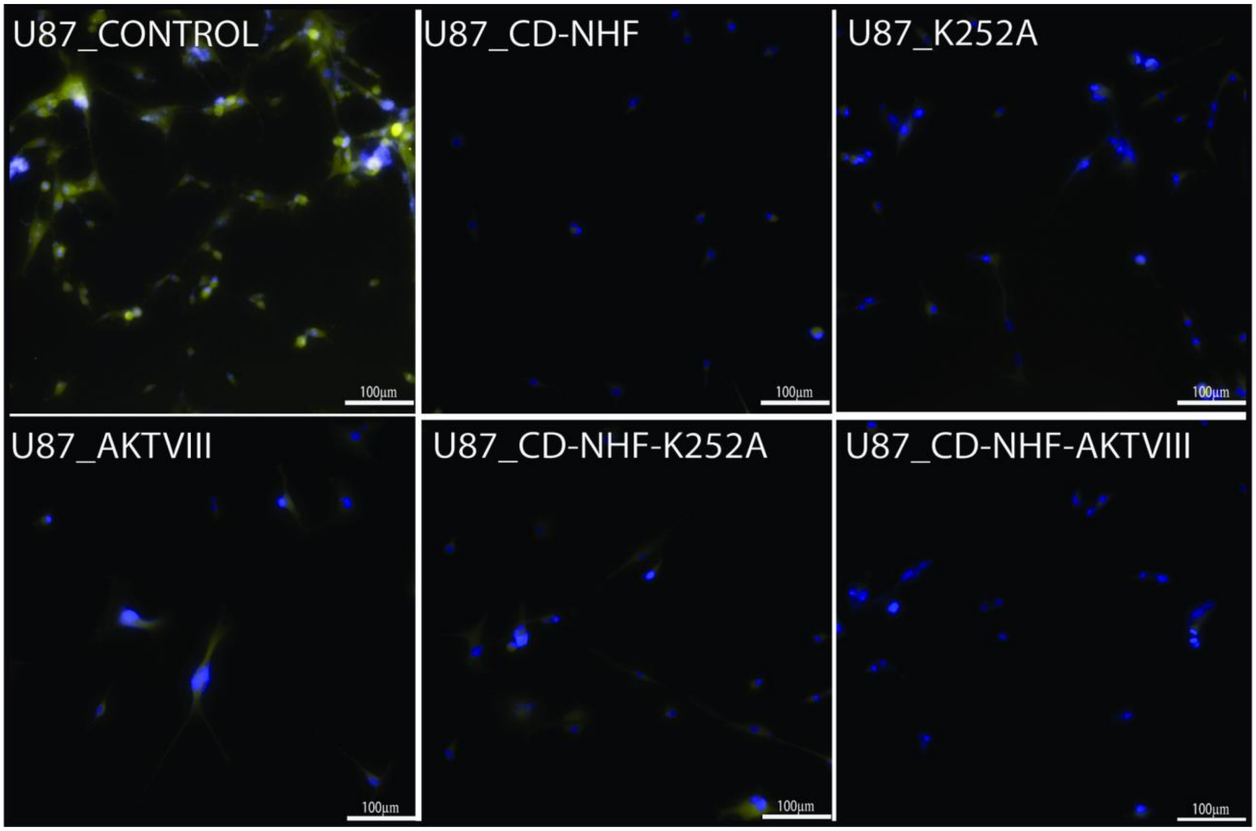

Figure S7. Representative images for immunofluorescence Bcl-2 staining. Merged stainings of Bcl-2 (yellow) and nuclei (blue). Pictures acquired at 20x.

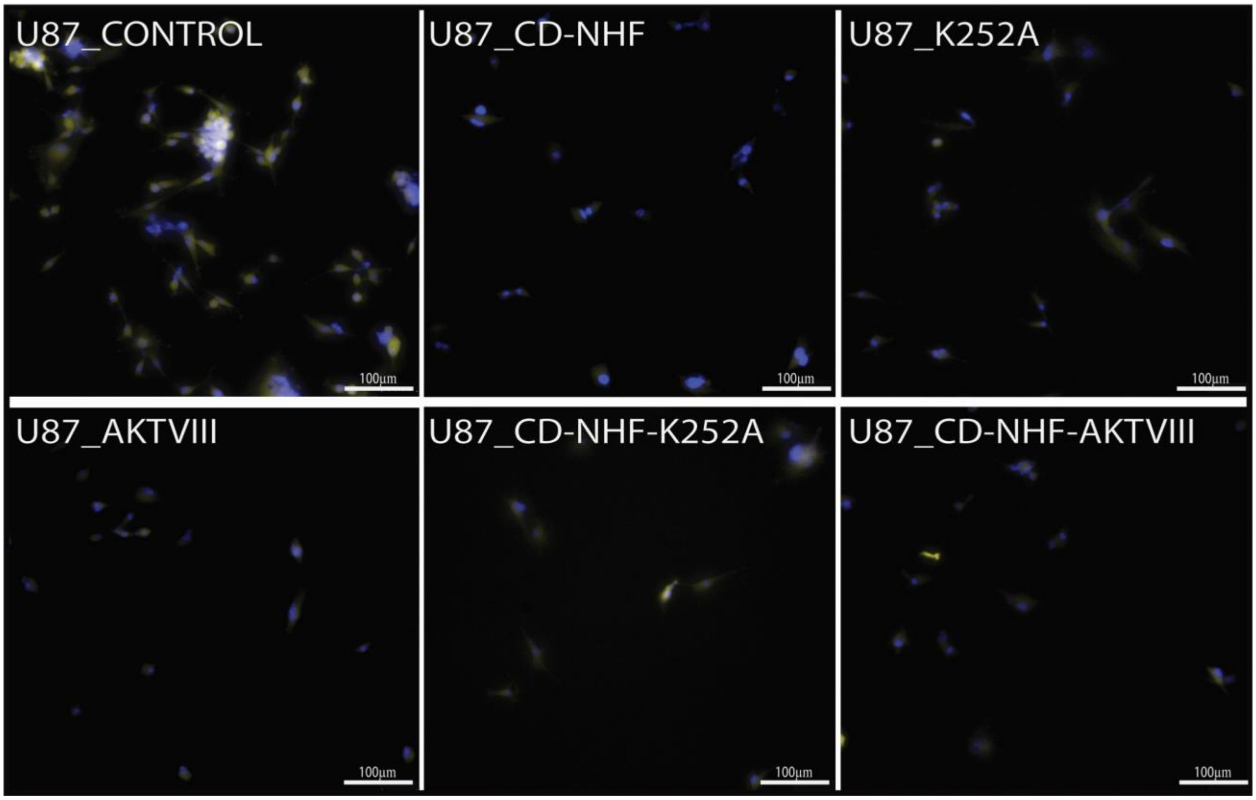

Figure S8. Representative images for immunofluorescence STAT3 staining. Merged stainings of STAT3 (yellow) and nuclei (blue). Pictures acquired at 20x.

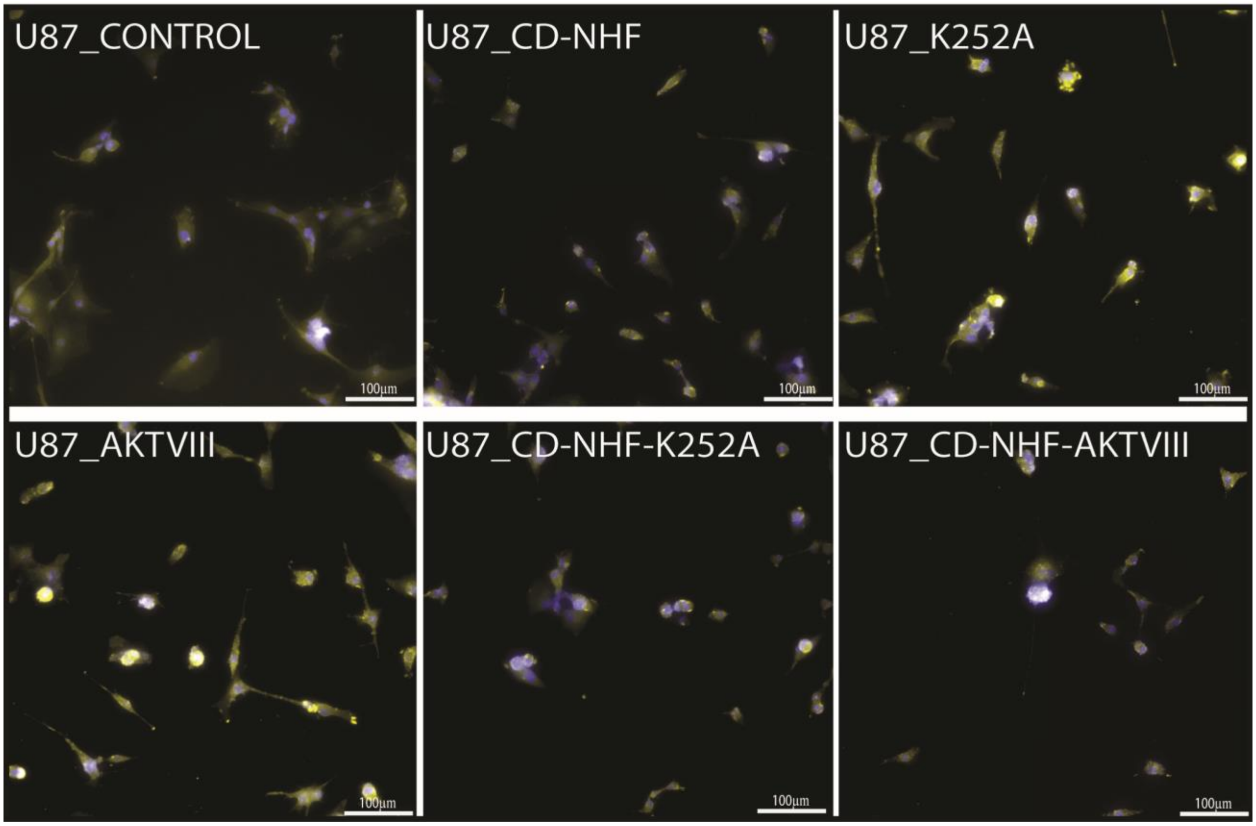

Figure S9. Representative images for immunofluorescence Slug staining. Merged stainings of Slug (yellow) and nuclei (blue). Pictures acquired at 20x.

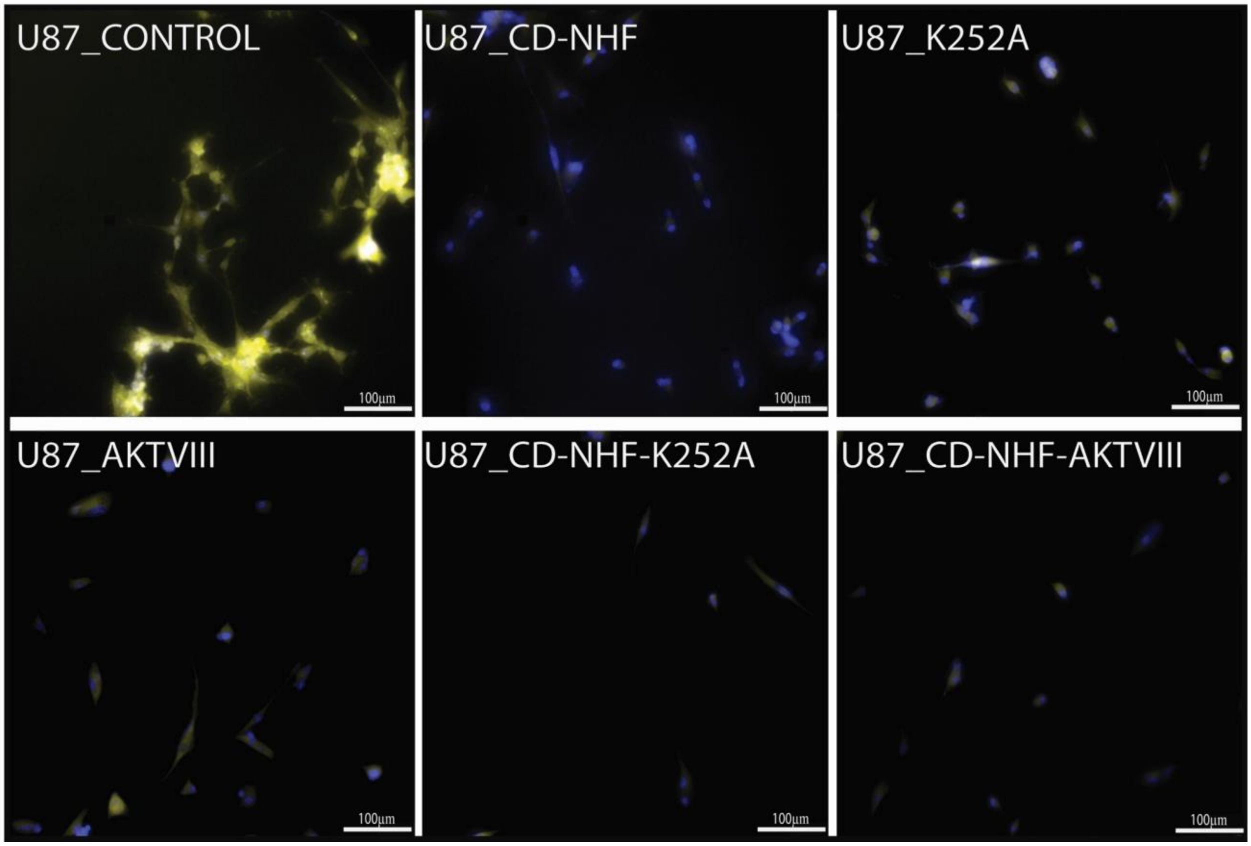

Figure S10. Representative images for immunofluorescence IL-6 staining. Merged stainings of IL-6 (yellow) and nuclei (blue). Pictures acquired at 20x.
